# Spatial, Social and Serological Factors in the Prevalence and Risk of Leprosy in Areas of High Endemicity: An Integrative Review

**DOI:** 10.3390/idr17030057

**Published:** 2025-05-22

**Authors:** Daniele dos Santos Lages, Isabela Cristina Lana Maciel, Sarah Lamas Vidal, Francisco Carlos Félix Lana

**Affiliations:** 1 Postgraduate Program in Nursing, School of Nursing, Federal University of Minas Gerais, Belo Horizonte 30130-100, MG, Brazil; daniele-lages@ufmg.br (D.d.S.L.); isabelaclm@ufmg.br (I.C.L.M.); sarahlamasvidal@ufmg.br (S.L.V.); 2 Department of Maternal and Child Nursing and Public Health, School of Nursing, Federal University of Minas Gerais, Belo Horizonte 30130-100, MG, Brazil

**Keywords:** leprosy, epidemiology, public health surveillance

## Abstract

Background/Objectives: Leprosy remains a global public health challenge, especially in hyperendemic areas, where spatial, socioeconomic and serological factors influence its persistence. In this study, an integrative review was carried out to analyze the relationship between these factors and the prevalence of *Mycobacterium leprae* infection, as well as the risk of falling ill. Methods: The integrative search was conducted in the BVS (Medline and LILACS) and Scopus databases, including studies published between 2010 and 2024; PRISMA was followed. Results: The findings indicate that spatial analysis, using geographic information systems, is essential for identifying transmission clusters and targeting control strategies. Poor socioeconomic conditions, such as low income and inadequate sanitation, significantly increase the risk of infection. In addition, serology, especially the detection of Anti-PGL-1 antibodies, has proved to be a promising tool for tracking subclinical infections and improving epidemiological surveillance. However, the integration of spatial, social and serological factors is still limited in the literature. Conclusions: We conclude that multidisciplinary approaches, combining spatial, socioeconomic and serological factors, are fundamental to optimizing control strategies and reducing leprosy transmission in vulnerable populations.

## 1. Introduction

Leprosy, a chronic infectious disease caused by the bacillus *Mycobacterium leprae* (*M. leprae*) [1], remains a significant public health problem in many parts of the world. Classified as a neglected disease by the World Health Organization (WHO), leprosy not only compromises the physical health of individuals, but also causes serious social and psychological impacts, due to the stigma associated with the condition [2,3,4,5,6]. Despite advances in diagnosis and treatment, there are still areas of high endemicity, where the infection persists and the rates of new occurrences remain high. Effective control of the disease is therefore a priority [7].

Leprosy has a clinical course that involves three main stages: exposure, infection and illness. In areas of high endemicity, a higher prevalence of infected individuals is expected, including those who have not yet developed clinical symptoms, but who can act as sources of transmission of the bacillus. The relationship between exposure, infection and illness is influenced by multiple factors, including spatial, socioeconomic and serological aspects, which are essential to understanding the epidemiological dynamics of the disease [8].

Thus, the prevalence of *M. leprae* infection is influenced by a complex interaction of factors that go beyond the biology of the pathogen [8]. Geographical factors, such as location and environmental characteristics, as well as population distribution, play a crucial role in the spread of the disease [9].

Spatial analysis using Geographic Information Systems (GIS) makes it possible to identify patterns of leprosy dispersion, facilitating the visualization of areas of greater risk and contributing to the targeting of public health interventions [10,11,12]. These approaches have the potential to support the development of more specific control policies, promoting the efficient use of available resources.

In addition to spatial factors, early detection of *M. leprae* infection is essential to reduce transmission and the physical disabilities associated with leprosy [13]. Serology, especially the detection of antibodies such as Anti-PGL-1, has proved to be an effective tool for assessing the burden of infection in populations, allowing the identification of asymptomatic cases that can act as vectors of the disease [13,14].

Studies suggest that the immunology and serological response of individuals vary according to socioeconomic and environmental factors, creating a diverse picture that should be considered in intervention strategies [15]. However, the relationship between these serological factors and the prevalence of leprosy still needs to be investigated further, highlighting a gap in the scientific literature [8].

In this context, this integrative review aims to identify, analyze and synthesize the existing literature on spatial, social and serological factors associated with the prevalence of *M. leprae* infection and the risk of falling ill with leprosy in areas of high endemicity. The risk of becoming ill is related to the clinical course of leprosy, so in hyperendemic areas, a higher rate of exposure and infection is expected, with the possibility of subclinical cases that can act as sources of transmission, making early identification and integrated control strategies essential.

By compiling and discussing the relevant findings, the aim is to contribute to the development of theories that explain the dynamics of the disease and to the formulation of more effective control and prevention strategies.

The review seeks to answer the central question: What is the association between spatial, social and serological factors and the prevalence of Mycobacterium leprae infection and the risk of falling ill with leprosy in areas of high endemicity? By addressing this question, we hope not only to contribute to the understanding of the epidemiological dynamics of leprosy, but also to elucidate gaps in current knowledge that can be explored in future research, as well as to provide a comprehensive overview of the state of the art in leprosy research, with an emphasis on the application of spatial, social and serological analyses.

The aim of this study is to identify, analyze and synthesize the existing literature on spatial, social and serological factors associated with the prevalence of *Mycobacterium leprae* infection and the risk of falling ill with leprosy in highly endemic areas.

## 2. Materials and Methods

The methodology of this study followed the principles of an integrative review [16], a research method that allows evidence on a given topic to be synthesized by combining data from empirical and theoretical literature. The article search and selection process included the “Biblioteca Virtual em Saúde—Virtual Health Library” (BVS) (Medline and LILACS) and Scopus databases selected for their relevance in the field of public health and epidemiology, covering literature published in the last 15 years (2010–2024) in English, Portuguese and Spanish.

Although PubMed is widely used for bibliographic reviews, we chose to carry out the search via Medline on the interface of the Virtual Health Library (VHL), which integrates PubMed records with other regional databases, such as LILACS. The BVS allows the use of multilingual descriptors (DeCS), broadening the coverage of Latin American literature and favoring the identification of studies relevant to the epidemiological context of leprosy in Brazil.

The design of the search strategy was guided by the review’s guiding question. Controlled and free terms related to leprosy, *Mycobacterium leprae* infection, serology (including Anti-PGL-1, MLFlow and antibody detection), spatial analysis (such as geographic information systems and cluster analysis), as well as social determinants (socioeconomic factors, poverty and sanitation) were used. The keywords were combined using Boolean operators suitable for each database to maximize the scope of the search (Table 1). The searches were carried out on 22 October 2024. Changes in the indexing of databases, updates to search algorithms or the automatic application of filters can cause searches carried out at different times to return different results. The studies analyzed in this review correspond to the set retrieved on that date, ensuring methodological consistency and traceability.

The inclusion criteria for the studies considered original articles that analyzed spatial, social and serological factors related to leprosy, including studies of prevalence and risk of illness in areas of high endemicity, such as Brazil and other tropical countries. Studies that did not specifically address the relationship between leprosy and the factors of interest or that did not present geospatial, social or serological data as the objects of the study were excluded. The main aim of this methodological decision was to identify gaps in the integrative literature on the subject.

After the search, the results were exported to the Rayyan (QCRI - Qatar Computing Research Institute; Doha, Qatar; Version - web app) platform, an online tool that facilitates the screening and selection of articles in literature reviews. The selection of studies was conducted according to the PRISMA (Preferred Reporting Items for Systematic Reviews and Meta-Analyses) 2020 methodology [17], ensuring rigor and transparency in the process. It is worth noting that, to date, there is no specific extension of PRISMA for integrative reviews, but its general guidelines were applied to structure the search and selection of studies. After excluding duplicates and applying the previously defined inclusion and exclusion criteria, the articles were analyzed in their entirety, ensuring that the studies included met the proposed objectives.

The selected articles were analyzed in terms of their methodological characteristics, the samples studied, the variables investigated and the main results. In addition, the analysis was conducted to identify the contributions of existing studies as well as the presence of gaps in the literature, especially with regard to the application of spatial analysis techniques and the use of serological markers in the early detection of *M. leprae* infection. The focus was on the spatial heterogeneity of leprosy in hyperendemic areas, and the impact of socioeconomic factors on the distribution and risk of illness.

In the end, the results of the integrative review were synthesized to show the state of the art on factors associated with the prevalence of infection and the risk of falling ill with leprosy, with emphasis on spatial, social and serological characteristics.

## 3. Results

**Figure 1:**
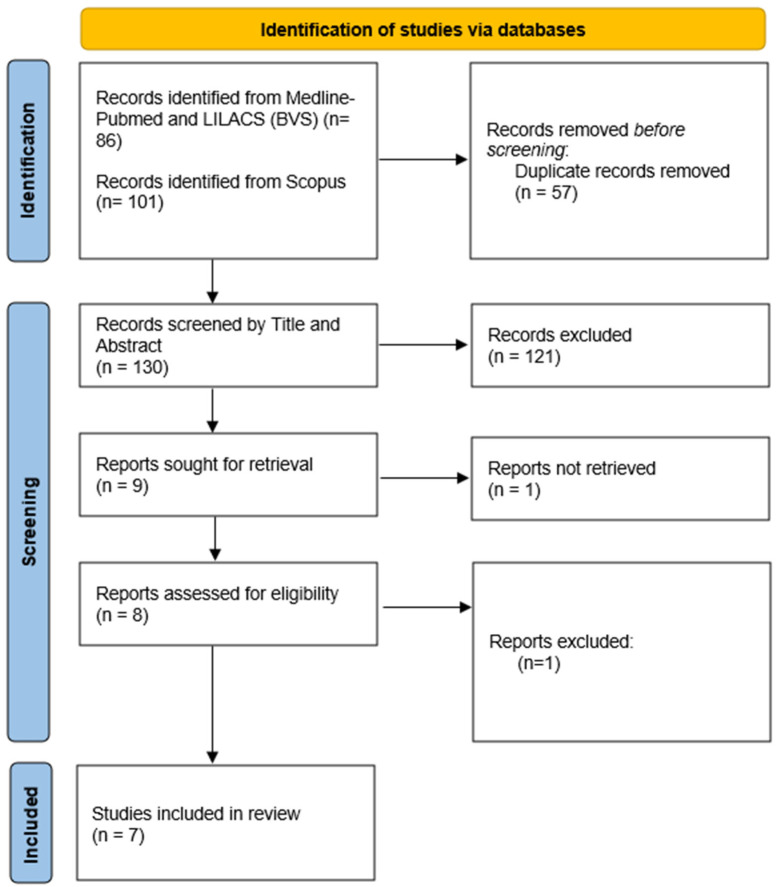
PRISMA flow chart: Process of selecting eligible studies for the review.

When searching the databases, 187 studies were retrieved, 57 of which were removed for being duplicates. A total of 130 titles and abstracts were evaluated, and of these, 9 studies were selected for reading the full text; however, 1 could not be retrieved (Figure 1).

This integrative review analyzed a total of seven articles addressing spatial, social and serological factors associated with the prevalence of *M. leprae* infection and the risk of falling ill with leprosy in areas of high endemicity. The data extracted from the studies are shown in Table 2.

Of the seven articles analyzed [18,19,20,21,22,23,24], published between 2010 and 2021 (Table 1), six focused on leprosy-endemic areas. The studies agree that spatial analyses, when combined with serological indicators, help in the process of understanding the dynamics of *M. leprae* transmission, allowing the identification of priority regions for intervention.

As for the identification of index cases in the studies analyzed, most used clinical diagnosis as a criterion, carried out by specialized professionals. Some studies included complementary laboratory tests, such as bacilloscopy or PCR, for confirmation.

Among the articles reviewed, three [18,19,21] emphasize the importance of spatial analysis tools in identifying clusters of high endemicity, enabling a more efficient allocation of resources in vulnerable areas. These studies emphasize that socioeconomic variables, such as high population density, low income and inadequate infrastructure, correlate positively with the risk of leprosy. The spatial heterogeneity observed reinforces the need to prioritize interventions in districts with poor infrastructure and high levels of poverty, where transmission of the disease is more intense.

In addition, three studies [20,22,23], highlight the importance of serological surveillance for detecting subclinical infections, with significant seropositivity rates among close contacts and specific groups within the community. The identification of asymptomatic carriers with high seropositivity suggests a silent source of transmission, and widespread serological testing is recommended as an essential tool to prevent new infections in areas of high endemicity.

In the context of household transmission, four studies [20,21,23,24] emphasize that close contacts of leprosy patients are at high risk of becoming ill, indicating the need for surveillance strategies focused on household networks and frequent social contacts. The proximity of seropositive contacts in households and the high incidence in areas with poor housing conditions suggest that preventive strategies should consider both the spatial dynamics and the social vulnerability of contacts.

Finally, ref. [24] emphasizes that the combination of serological and spatial analyses allows for a more precise approach when identifying risk areas and adapting interventions to the particularities of local populations. This integrated approach facilitates continuous surveillance and more effective control of leprosy in contexts of high endemicity, highlighting the need for policies that combine serological surveillance with territorial analysis.

## 4. Discussion

The integrative review aimed to identify and analyze the spatial, social and serological factors associated with the prevalence of *M. leprae* infection and the risk of falling ill with leprosy in areas of high endemicity, as well as pointing out contributions and gaps in existing knowledge. The central guiding question sought to understand how these factors influence the epidemiological dynamics of leprosy and how they could be explored in public health interventions and future research.

Studies analyzed [18,19,21] show that spatial analysis is a powerful tool for identifying areas at greater risk of leprosy. By applying geographic information systems (GIS) and spatial statistical methods, it was possible to delineate hyperendemic regions and identify transmission hotspots. These findings allow for more targeted interventions, both in the prevention and control of the disease, maximizing the impact of public health policies in these regions. The identification of spatial patterns in areas with high prevalence, especially in places with greater socioeconomic vulnerability and poor health infrastructure, demonstrated the importance of targeting resources and control strategies to the most affected areas.

Furthermore, the correlation observed between socioeconomic factors, such as high population density, poverty and inadequate infrastructure, reinforces the need for targeted interventions in these areas [18,19,21]. Combining spatial analysis with socioeconomic data allows resources to be allocated more efficiently, especially in districts with high prevalence and poor infrastructure, where disease transmission is more intense [18,19,20].

In the context of household transmission, studies such as those by [21,24] have highlighted that family contacts of leprosy patients are particularly vulnerable to infection. This pattern of intra-household transmission was identified as one of the main sources of maintaining the chain of infection, especially in hyperendemic areas, reinforcing the need for active and specific epidemiological surveillance to this group. These findings underline the importance of contact tracing strategies, continuous monitoring and early interventions to stop the spread of the disease.

Serological factors have also been identified as fundamental in the control of leprosy. Studies such as those by [20,23] have shown that the use of serological markers, such as anti-PGL-I and RLEP, can reveal subclinical infections in asymptomatic individuals. These silent carriers of *M. leprae* represent an additional challenge for disease control, as they can contribute to transmission even without clinical symptoms. This is because individuals with simultaneous serological and molecular positivity are more likely to be subclinical transmitters [13,23]. Although the extent of their role in disease transmission is still being investigated, this group has been increasingly considered in surveillance and chemoprophylaxis strategies in hyperendemic areas.

The combination of serological and spatial approaches, as shown by [24], proved to be particularly promising. By integrating serological surveillance with spatial analysis, it was possible to identify areas with a higher concentration of subclinical infections and more vulnerable population groups, allowing for earlier and more targeted interventions. This integrated approach not only facilitates the early identification of infections, but also allows for the more efficient allocation of health resources and the formulation of more effective interventions focused on the areas of greatest vulnerability.

However, despite the significant contributions of these studies, a knowledge gap persists in research. Despite the demonstrated effectiveness spatial and serological surveillance, most of the studies analyzed did not extensively consider serological markers, such as anti-PGL-I, and their relationship with social and environmental factors. This limitation prevents a more comprehensive understanding of the hidden sources of transmission and the epidemiological dynamics of leprosy in areas of high endemicity.

This gap suggests the need for future research that explores integrated models, combining spatial and serological aspects with environmental and socioeconomic factors, to provide a more complete and multidimensional view of the epidemiology of leprosy. In addition, there is a need to further investigate how social conditions and the structure of family and community networks can influence vulnerability to becoming ill and the spread of the disease.

Recent studies reinforce this need by demonstrating that the combination of spatial analysis and serology, especially the detection of anti-PGL-I antibodies, can improve the identification of areas and groups most at risk, contributing to more targeted leprosy control strategies [25]. In addition, social determinants continue to be critical factors in maintaining the endemic, requiring intersectoral approaches to mitigate their influences.

## 5. Conclusions

The results of this integrative review highlight the importance of an integrated approach between spatial, social and serological factors to understand the dynamics of leprosy in areas of high endemicity. Spatial analysis proved to be essential for identifying transmission hotspots, enabling more efficient targeting of interventions. At the same time, socioeconomic factors proved to be critical determinants for maintaining the endemic, reinforcing the need for intersectoral strategies aimed at improving the living conditions of the affected population. Serology, meanwhile, has emerged as a valuable tool for detecting subclinical infections, helping to trace contacts and control transmission.

However, this review highlights a relevant gap in the scientific literature: the scarcity of studies that simultaneously integrate spatial, social and serological factors in the analysis of leprosy. This finding reinforces the importance of interdisciplinary approaches in the surveillance and control of the disease.

The integrated analysis of infection markers, social determinants and spatial distribution can support a more refined and structured form of epidemiological surveillance, capable not only of monitoring clinical cases, but also of anticipating transmission patterns and identifying vulnerable populations before they become ill. This approach contributes to more timely and targeted public health responses, which is essential for reducing the leprosy burden in areas of high endemicity.

It is worth noting that all the studies found that met the inclusion criteria were carried out in Brazil. This concentration reflects the significant burden of the disease in the country, which is second in absolute number of cases worldwide. However, the social and spatial heterogeneity of Brazil favors the extrapolation of the findings to similar endemic contexts.

It is therefore recommended that future research prioritize methodologies that combine spatial, socioeconomic and serological analyses, contributing to more effective leprosy control. In addition, public policies should incorporate this integrated approach, promoting coordinated actions between epidemiological surveillance, social assistance and early diagnosis to mitigate the impact of the disease on vulnerable populations.

## Figures and Tables

**Table 1:** Search strategy.

**Virtual Health Library (BVS—Medline and LILACS)**	(“Leprosy” OR “Leprosy” OR “Lepra” OR “Mycobacterium leprae”) AND (“Prevalence” OR “Prevalencia” OR “Infection rate”) AND (“Serology” OR “Serologia” OR “Serología” OR “Anti-PGL-1” OR “ML Flow”) AND (“Risk of becoming ill” OR “Riesgo de enfermedad” OR “Disease risk” OR “Incidência” OR “Incidence” OR “Incidencia” OR “Susceptibility” OR “Susceptibilidad”) AND (“Análise espacial” OR “Análisis espacial” OR “Spatial analysis” OR “Sistema de Información Geográfica” OR “Geographic Information System” OR “Cluster analysis” OR “Análisis de conglomerados” OR “Spatial autocorrelation” OR “Autocorrelación espacial” OR “Spatial patterns” OR “Patrones espaciales”) AND (“Determinantes sociais” OR “Determinantes sociales” OR “Social determinants” OR “Factores socioeconómicos” OR “Socioeconomic factors” OR “Pobreza” OR “Poverty” OR “Saneamiento” OR “Sanitation”)
**Scopus**	(“Hanseníase” OR “Leprosy” OR “Lepra” OR “Mycobacterium leprae”) AND (“Prevalência” OR “Prevalence” OR “Prevalencia” OR “Infection rate”) AND (“Serology” OR “Serologia” OR “Serología” OR “Anti-PGL-1” OR “ML Flow”) AND (“Risk of illness” OR “Riesgo de enfermedad” OR “Disease risk” OR “Incidência” OR “Incidence” OR “Incidencia” OR “Susceptibility” OR “Susceptibilidad”) AND (“Análise espacial” OR “Análisis espacial” OR “Spatial analysis” OR “Sistema de Información Geográfica” OR “Geographic Information System” OR “Cluster analysis” OR “Análisis de conglomerados” OR “Spatial autocorrelation” OR “Autocorrelación espacial” OR “Spatial patterns” OR “Patrones espaciales”) AND (“Determinantes sociais” OR “Determinantes sociales” OR “Social determinants” OR “Factores socioeconómicos” OR “Socioeconomic factors” OR “Pobreza” OR “Poverty” OR “Saneamiento” OR “Sanitation”) AND PUBYEAR > 2009 AND PUBYEAR < 2025 AND (LIMIT-TO (DOCTYPE, “ar”)) AND (LIMIT-TO (LANGUAGE, “English”) OR LIMIT-TO (LANGUAGE, “Portuguese”) OR LIMIT-TO (LANGUAGE, “Spanish”)) AND (LIMIT-TO (PUBSTAGE, “final”)) AND (LIMIT-TO (OA, “all”)))

Source: prepared for the purposes of this study. Database search carried out on 22 October 2024.

**Table 2:** Characteristics of the studies included in the integrative review.

Title	Authors	Year	Methodological Approach	Key Results	Contribution of the Study to Answering the Research Question
Geographic information systems and applied spatial statistics are efficient tools to study Hansen’s disease (leprosy) and to determine areas of greater risk of disease.	[18]	2010	Cluster analysis and GIS	It identified two high-risk areas with statistical significance, with a relationship between the distribution of leprosy and socioeconomic variables of poverty. The use of GIS and spatial analysis proved effective in mapping clusters of the disease, indicating areas for targeting control interventions.	It contributes by identifying high-risk areas and correlating them with socioeconomic factors, supporting specific interventions in the regions most affected by leprosy.
Hot spots of leprosy in the endemic area of São Luís, Maranhão state, northeastern Brazil	[19]	2019	Spatial analysis and socioeconomic indicators	It found positive correlations between cases and the number of residents, open sewers and low income, while sidewalks and schooling had a negative correlation. Seropositivity among contacts was 23.19%. It has a heterogeneous spatial pattern, with hyperendemic clusters.	The study advances the understanding of the social and spatial factors that influence the prevalence of leprosy, showing the importance of infrastructure and socioeconomic conditions in transmission.
Widespread nasal carriage of Mycobacterium leprae among a healthy population in a hyperendemic region of northeastern Brazil	[20]	2015	Cross-sectional study	The prevalence of RLEP positivity was high among cases (69.2%) and contacts (66.9%), with dissemination of the bacillus among the healthy population. Factors associated with leprosy included male gender, low income, previous contact and age. It defined areas of multibacillary case clusters.	It reinforces the importance of molecular and social factors, suggesting surveillance of contacts and endemic areas to control transmission in vulnerable communities.
Spatial distribution pattern of new leprosy cases under 15 years of age and their contacts in Sobral, Ceará, Brazil	[21]	2021	Spatial analysis and household contact	It detected leprosy cases in children under 15 in hyperendemic areas, with higher seropositivity rates among household contacts. It identified clusters of subclinical infection in low-income areas, suggesting that transmission is related to the proximity of seropositive individuals.	It highlights the role of social factors and physical proximity in transmission, contributing to interventions in vulnerable families and areas.
Spatial and temporal epidemiology of Mycobacterium leprae infection among leprosy patients and household contacts of an endemic region in Southeast Brazil	[22]	2016	Serology (anti-PGL-I and NDO-LID) and spatial analysis	It observed high seropositivity (42%) in contacts for the NDO-LID rapid test, with three groups of subclinical infection identified. The spatial and serological evaluation proposed priority areas for surveillance and prevention.	It contributes by linking serology and spatial analysis, proposing targeted surveillance in high-risk areas, strengthening leprosy control in endemic locations.
Latent leprosy infection identified by dual RLEP and anti-PGL-I positivity: Implications for new control strategies	[23]	2021	Serology (PGL-I) and molecular analysis (RLEP) in multiple groups	He found high seropositivity (46%) for contacts with active leprosy, with a higher risk of progression to clinical disease. He suggested chemoprophylaxis for double-positive household contacts to reduce transmission.	It reinforces the importance of identifying serologically and molecularly positive contacts to prevent the spread of leprosy in endemic areas.
Combined use of serological markers and spatial analysis in the epidemiological surveillance of leprosy	[24]	2021	Serology (anti-PGL-I) and territorial analysis	Seropositivity was higher among family members and neighbors of positive schoolchildren. It found a higher rate in low-income households and areas with few rooms. The territorial analysis indicated a possible hidden endemic and identified vulnerable sectors for active screening.	It integrates serological and spatial factors, suggesting screening activities for early diagnosis and control in areas with a high concentration of seropositivity.

## Data Availability

Data can be made available by the authors upon request.

## Abbreviations

The following abbreviations are used in this manuscript:

WHO	World Health Organization
*M. leprae*	*Mycobacterium leprae*
GIS	Geographic Information Systems
QCRI	Qatar Computing Research Institute
PRISMA	Preferred Reporting Items for Systematic Reviews and Meta-Analyses
